# The effects of challenge or social buffering on cortisol, testosterone, and antler growth in captive red deer (*Cervus elaphus)* males

**DOI:** 10.1038/s41598-023-48476-9

**Published:** 2023-12-09

**Authors:** Luděk Bartoš, Bruno Esattore, Radim Kotrba, Jan Pluháček, Francisco Ceacero, Martina Komárková, Adam Dušek, Jitka Bartošová

**Affiliations:** 1 Department of Ethology, Institute of Animal Science, Přátelství 815, Praha Uhříneves, 10400 Czech Republic; 2 Department of Game Management and Wildlife Biology, Faculty of Forestry and Wood Sciences, Czech University of Life Sciences, Kamýcká 129, 16521 Praha 6, Czech Republic; 3 Faculty of Tropical AgriSciences, Czech University of Life Sciences Prague, Kamýcká 129, 16521 Praha 6, Czech Republic

**Keywords:** Sexual selection, Social evolution, Zoology, Endocrinology

## Abstract

We equipped 17 captive red deer males (*Cervus elaphus*) with GPS collars to measure inter-individual distances throughout the 5-months of the antler growth period. We expected some individuals to associate regularly with others while others would not. We predicted that males aggregating with others within a socially stable environment (Associates) would benefit from a form of “social buffering” and would likely have lowered cortisol (C) and testosterone (T) concentrations. Males only irregularly joining social groupings would experience elevated levels of aggression; according to the “Challenge hypothesis”, their T and C concentrations should increase. Interacting with a higher proportion of Associates did indeed reduce C concentrations. Conversely, avoiding Associates and challenging other males stimulated the T secretion. Admittedly, males avoiding regular proximity to others tended to develop the largest antlers. They probably benefited from frequent successful agonistic threats to conspecifics, resulting in elevated T concentrations. Regular association with tolerant, conspecifics and “social buffering” did not seem sufficient for producing larger antlers despite reducing C concentrations. Alternative social strategies were adopted within the same group of individuals and showed how the trade-off between these strategies could have an essential impact on C and T concentrations.

## Introduction

Mammalian societies are complex systems, influenced and modified by numerous factors, both external and internal. Among the latter, inter-individual relationships play a key role in shaping social systems^1^. Two different types of relationships can exist within mammalian social systems: dominance relationships and social bonding, which are established and maintained by socio-positive behaviours^2^. Over the last few decades, extensive work has been undertaken on social and dominance relationships in several deer species, especially in the situation where males live in a bachelor group, and the inter-individual relationships can affect antler growth and formation^3–5^. Analysis of the grouping dynamics of red deer males under natural conditions throughout the antler growth period shows that individuals tended to associate with others of similar rank and age^6,7^. However, such associations are not necessarily permanent; groupings may disintegrate, and some individuals may remain solitary. The type of grouping in which males may associate and the social stimuli experienced as a consequence has a clear effect on an individual's physiological responses, including antler growth^3,4^, timing of antler casting^8^ and cleaning in red deer^8^ and actual antler development in red^9^ and fallow deer *Dama dama*^10^. Antlers are an important trait in sexual selection in cervid species (e.g., in red deer^11,12^). Thus, any tactic leading to changing antler size has the potential to affect reproduction success and, hence, the male's reproductive fitness.

Generally, among social mammal species, dominant individuals were thought to increase physiological stress in subordinates, while reducing stress in themselves (e. g.,^13,14^). However, a number of studies seemed to break such a rule^2,15–17^. Social behaviour has been shown to affect and be affected by several different hormones, with the best-established connections being steroid hormones^18^. Among them, testosterone (T) and cortisol (C) play a fundamental role in the life of deer. Thus, T promotes and regulates antler growth and cycle timing in males^3^, while C is the hormone mediating significant energy mobilisation and redistribution in the face of enhanced physical activity, as well as responses to a stressful situation^19^.

The Hypothalamus-pituitary–gonadal axis (HPG) regulates T concentrations, while the Hypothalamus–pituitary–adrenal axis (HPA) processes C. Elevated levels of C can inhibit T secretion^20,21^. C and T thus do not act independently when influencing dominance and competition (e.g.,^22^) since the activation of one affects the function of the other (e.g.,^23^). Increasing T requires increasing C to fulfil the energy resources. However, in order to reach levels required to meet environmental demands C concentrations may exceed the natural regulatory capacity of the organism, resulting in stress^19^. High levels of C in turn suppress concentration of T, while the suppressive effect of T on C may lead to a stage of reduced recovery of the neuroendocrine reaction^19^. That is why both hormones should work in synergy to guarantee an adequate supply of energy resources to the organism^23^.

When studying the relationships between dominance-related behaviour and T and C concentrations which result, researchers have predominantly focused on aggressive behaviour in a variety of animal species^15,24–27^. In a previous study on red deer, the introduction of nine 3-year-old males into a socially stable group of 12 mature males resulted in a change of the relationship between rank and both T and C concentrations. Adult males preferentially targeted their attacks to individuals much lower in the hierarchy and reduced the frequency of frustrating interaction with other adults, despite the fact that the rank positions of the adults did not change^15^. In this study we have focused specifically on how an individual male perceives its social position within a given group, and included two considerations that have not been considered in previous studies investigating the relationship between social behaviour and physiological responses affecting antler growth.

The first aspect is that of “social buffering” (“the buffering hypothesis^28–31^”) meaning the ability of association with a social partner to mitigate potential stress responses^30,32^. The presence of a close social partner attenuates the reactivity of the HPA and thus buffers against the potentially adverse effects of physiological stress^33^. Taylor and Master^34^ termed such affiliative responses “tend-and-befriend”. The individual functioning as a social buffer against stress^28,30,32^ may be a pair-bonded female partner (e.g., in humans^35^). This is less common in non-human animals^30,33,36^, where affiliative bonds are more frequent among individuals of the same sex. Recent and increasing evidence has shown that primate males may regularly form strong social bonds that can enhance their fitness e. g.,^33,37–40^. Clear demonstration of a stress-ameliorating effect of non-kin social bonding among males in natural or semi-natural situations is however very rare, with the only exception being among Barbary macaques (*Macaca sylvanus*)^33^.

In horses, elephants, hyenas, dolphins, and several primate species, some individuals form friendships that may last for years, and many friendships are formed between unrelated individuals^33,41,42^. Hence, we expected the same in gregarious species of deer. In red deer males (unlike females), social reinforcement behaviours (e.g., mutual grooming) is rarely observed. Therefore, we regarded as a potentially “friendly” behaviour when two males spent prolonged time close together without attacking each other. However, time spent closely together did not prevent such associating individuals from agonistic interactions to keep dominance relationships or compete for food^43^. We can only speculate that the encounters between such partners are not as challenging as those with males showing less obvious association. Thus, we considered the frequency of interactions with others in prolonged proximity to the focal individual as an indication of preferred association rather than escalated hostility.

A second concept we explored in evaluation of the effect of male-male competition during the period of antler growth, was a broader application of the “Challenge hypothesis”^44,45^. The authors of the original Challenge hypothesis proposed that the relationship between T concentrations in the blood and aggressive social behaviours should have a strong influence on circulating androgen levels across taxa^46,47^, especially among males^16^. Specifically, males should respond to social challenges from conspecific males with a rapid increase in plasma androgen levels, to stimulate and support further aggression^48^. In our study, we extended the original application of the Challenge hypothesis and focused on the possible elevation of T concentrations due to male-male competition during the non-breeding season. Such a situation might occur, for example, when the rank position of a male within the group is challenged by another male. We also expected that if such a challenging situation occurred repeatedly and consistently, it would increase mean T concentration over that entire period.

T has been found as a major hormone regulating antler growth^4,5^. Therefore, to develop the largest antlers, a male deer would face a trade-off between achieving the highest protection against stress or reaching the highest possible T concentrations to enhance antler growth. According to the buffering hypothesis^28^, it would be advantageous for such a male to be sociable, spending prolonged time close together with other individuals not attacking each other, thus minimizing social stress. This should result in lower C concentrations, but would not necessarily suppress T concentrations^3^. An alternative tactic would be avoiding a socially stable grouping, preventing the male from establishing a long-lasting relationship with others during the period of antler growth. Whenever such a male would meet other males then, an encounter would be challenging, because its social status would be threatened^16,44,46^. As a result, T concentrations should be increased, and antler growth should be greater unless elevated C concentrations would be suppressive to the elevated T concentrations.

We predicted that during the period of antler growth (i) some males would keep company with others for a long time, while others would not. This would suggest two different tactics, keeping together and keeping apart; (ii) when keeping together and aggregating with other tolerant males, the males would conform to social buffering, and an individual male would likely benefit from lowered C and T concentrations. When (iii), on the contrary, males tend to be more solitary in habit, only irregularly joining socially-unstable groupings, levels of aggression would likely be elevated, especially in high-ranked individuals. Consequently, T concentrations should increase. It would be expected that C concentrations would also increase, but not to an extent which would suppress the elevated T concentrations. Finally (iv), higher dominance status would be expected to reduce C concentrations, meaning dominant animals would have lower C and raised T as previously described in red deer ^3^. These predictions are summarized in Table 1. For testing such predictions, the red deer is an unusually suitable model. This is because the males grow antlers and antlers are an important trait in sexual selection. Therefore, antler size was measured to evaluate which social strategy would be more advantageous in producing larger antlers (and thus presumed reproductive fitness).

**Table 1:** Presumptions for the hypotheses advanced.

Hypothesis	Clustering into distinguishable groups	Consistently keeping together	Consistently keeping apart	Mean cortisol concentration	Mean testosterone concentration	Supportive reference
(i) An existence of alternative strategies	Yes	Yes	Yes	N/A	N/A	^43^
(ii) Social buffering	Yes	Yes	No	Low	Low	^28,30,32^
(iii) Challenge hypothesis	Yes	No	Yes	High	High	^16,45^
(iv) Dominance effect	Yes/No	Yes/No	Yes/No	Low	High	^3^

## Results

(Prediction i) Although the males had known each other for an extended period before we started our observation, they either associated with or separated from each other consistently throughout five months (April–August) in the main intensive study period. They continued in this pattern despite aggregating frequently when attracted by supplementary food, and breaking the average inter-individual distances for the time of feeding. Cluster analysis divided the dyadic average distances between males into two convincingly well-separated clusters of relationships of the interactions between the focal male and an interacting conspecific: “Associates” (number of dyads, mean ± SE, n = 108, 12.64 ± 0.49 m; Lower 95% CL for mean, Upper 95% CL for mean 11.66 m, 13.61 m) and “Distant” (n = 164, 141.60 ± 4.21 m, 133.30 m, 149.91 m). After the division of Distant dyads according to whether they interacted with others or not, the average inter-individual distances between distant males which did associate with other males but not with the focal male in any analysis (“Non-Associates”, n = 93) was 145.46 ± 5.69 m (Lower CL, 134.16 m, higher CL 156.76 m) and for distant non-interacting conspecifics (“Indifferent”, n = 71) 136.55 ± 6.22 m, (124.14 m, 148.96 m).

There were substantial differences between individuals in the proportion of interactions with Associates and Non-Associates over supplementary food (Table 2). Only two males had no interaction with an Associate and interacted only with Non-Associates. All males but two had no contact with some specific individuals (i. e., Indifferent, ranging from 0 to 13 other stags with no contact).

**Table 2:** List of potential fixed factors (mean, standard error, lower and upper 95% confidence limits, minimum, maximum per male) for each subject. Terms in square brackets are abbreviations used in defining a statistical model (n = 17).

Variable	Mean	Std Error	Lower 95% CL for Mean	Upper 95% CL for Mean	Min	Max	N
Age (years) [Age]	4.82	0.12	4.59	5.05	2.00	9.00	17
Mean cortisol concentration (ng/ml)* [Cortisol]	114.09	2.39	109.39	118.80	54.38	176.24	17
Mean testosterone concentration (ng/ml)* [Testosterone]	0.48	0.01	0.45	0.51	0.28	1.14	17
Total antler length (cm) [Tot_Antler_Length]	317.12	10.34	296.77	337.47	10.00	560.50	17
Number of attacked conspecifics [Number_attacked]	8.65	0.30	8.06	9.23	0.00	16.00	17
Sum of winning encounters [Wins]	56.71	3.02	50.76	62.65	0.00	147.00	17
Number of lost encounters [Losses]	56.71	2.20	52.38	61.03	4.00	118.00	17
Sum of all agonistic interactions (attacked others and being attacked) [Sum_interact]	113.41	3.07	107.36	119.47	17.00	181.00	17
Proportion of Associates of all dyadic relationships (%) [Proc_Ass]	39.71	1.67	36.42	42.99	0.00	75.00	17
Proportion of Non-Associates of all dyadic relationships (%) [Proc_NAss]	60.29	1.67	57.01	63.58	25.00	100.00	17
Body weight at the beginning of the observation period (kg) [Weight1]**	109.12	1.70	105.77	112.47	50.00	152.00	17
Body weight at the end of the observation period (kg) [Weight2]	142.12	2.60	137.01	147.23	42.00	196.00	17
Body weight gain over the period of observation (kg)**	33.00	1.28	30.48	35.52	− 8.00	77.00	17
Relative body weight gain over the period of observation (%)**	20.44	0.83	18.81	22.08	− 19.05	39.29	17
Mean blood sampling order* [Order]	10.42	1.26	7.74	13.09	3.00	17.00	17
Variable	Levels						
Dominance	Dominant, indifferent, subordinated						

*Mean value over the period of observation.

**Due to the high correlation with Weight 2, we did not use this variable in the a priori models.

Prediction (ii) In relation to factors influencing log-transformed C concentrations, the Supplementary Table S1 shows the five best candidate models ranked by the five criteria of best fit. All criteria ranked the same GLMM as the best. They did not differ in ranking the other candidate best models (Supplementary Table S1 top). Also the differences (Δ) between the best and second-best model were the same by all the criteria (Δ for second model, Δ AIC = 8.96, Δ AICC = 8.96, Δ BIC = 8.96; Δ CAIC = 8.96, Δ HQIC = 8.96).

By comparing our best model to the null model, we have a convincing argument that the best model has merit with apparently negligible information loss estimated by all five fit criteria (Supplementary Table S2). Since fitting by all criteria was similar, we present further calculations for AIC only. Table 3 shows five best fitting models sorted according to fit AIC (the smaller, the better), AIC difference (Δ_*i*_), AIC weight (*wi*), and AIC Odds for the dependent variable log-transformed C concentrations. The correct model's probability was high (99%) in comparison to the second best model (0.01%). The best fitting GLMM was thus 88.21 times (odds) more likely to be the correct model than the second best model.

**Table 3:** Five best-fitting models sorted according to fitting AIC (the smaller, the better), AIC difference (*Δ*_*i*_), AIC weight (*w*_*i*_), and AIC Odds for the dependent variables log-transformed Cortisol concentrations, log-transformed Testosterone concentrations, and Total antler length ("t" at the end of the effect's name in a model means "log-transformed").

Model	AIC	Δ_*i*_	*w*_*i*_	AIC Odds
For the dependent variable cortisolt				
Proc_Asst testosteronet Weight2t order	25.32	0.00	0.99	1.00
Proc_Asst testosteronet Weight2t dominance Order	34.28	8.96	0.01	88.21
Proc_Asst Testosteronet number_attacked order	54.81	29.49	0.00	2,536,242.79
Proc_Asst Testosteronet number_attacked dominance order	63.31	37.99	0.00	177,399,024.01
Proc_Asst testosteronet tot_INTERACT order	68.86	43.54	0.00	2,844,675,225.07
For the dependent variable testosteronet				
Proc_NAsst cortisolt losses order	− 461.47	0.00	0.84	1.00
Proc_NAsst cortisolt Tot_INTERACT order	− 456.98	4.49	0.09	9.44
Proc_NAsst cortisolt losses dominance order	− 455.48	5.99	0.04	19.96
Proc_NAsst cortisolt age order	− 453.45	8.02	0.02	55.13
Proc_NAsst cortisolt order	− 452.89	8.58	0.01	72.82
For the dependent variable total antler length				
Proc_NAsst age number_attacked Testosteronet Order	2793.27	0.00	0.96	1.00
Proc_NAsst age number_attacked Testosteronet	2799.48	6.21	0.04	22.27
Proc_NAsst age number_attacked Cortisolt Order	2817.00	23.73	0.00	142,005.64
Proc_NAsst age number_attacked Cortisolt	2825.37	32.09	0.00	9,309,289.14
Proc_Asst age number_attacked Cortisolt order	2825.83	32.55	0.00	11,723,985.44

**Figure 1:**
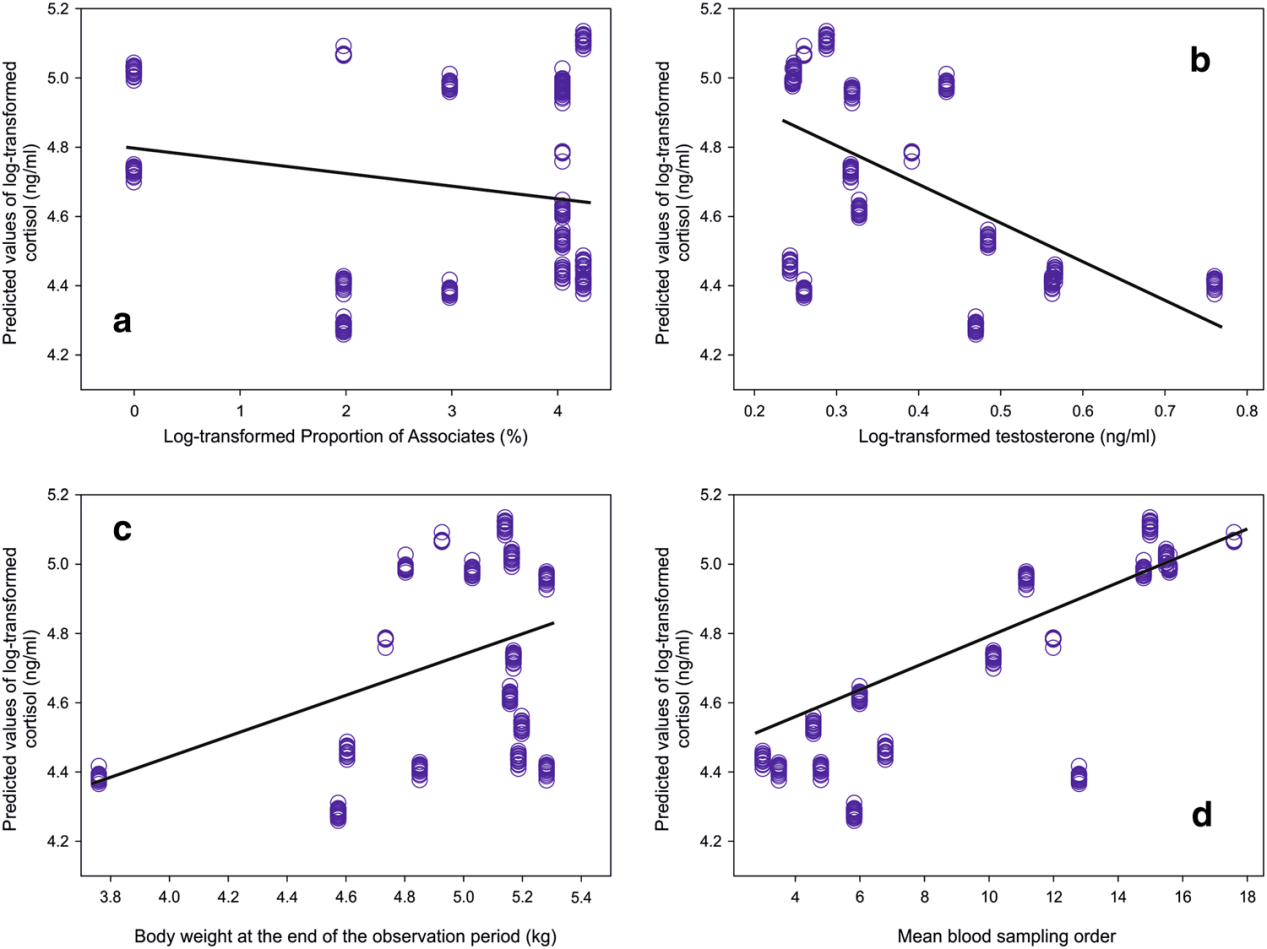
Predicted values of log-transformed cortisol concentrations (ng/ml) plotted against log-transformed Proportion of Associates (%) (**a**), log-transformed testosterone concentrations (ng/ml) (**b**), log-transformed body weight (kg) (**c**), and Mean blood sampling order (**d**).

According to the best model, C concentrations were affected by proportion of Associates (Fig. 1a, C concentrations decreasing with the increasing proportion of Associates), T concentrations (Fig. 1b, C concentrations decreased with the increasing T concentrations), by body weight at the end of the observation period (Fig. 1c, higher C concentrations with increasing body weight of the subject), and mean blood sampling order (Fig. 1d). Estimates, Standard error and 95% confidence interval for best fitting GLMM model for the C are presented in the Supplementary Table S3.

**Figure 2:**
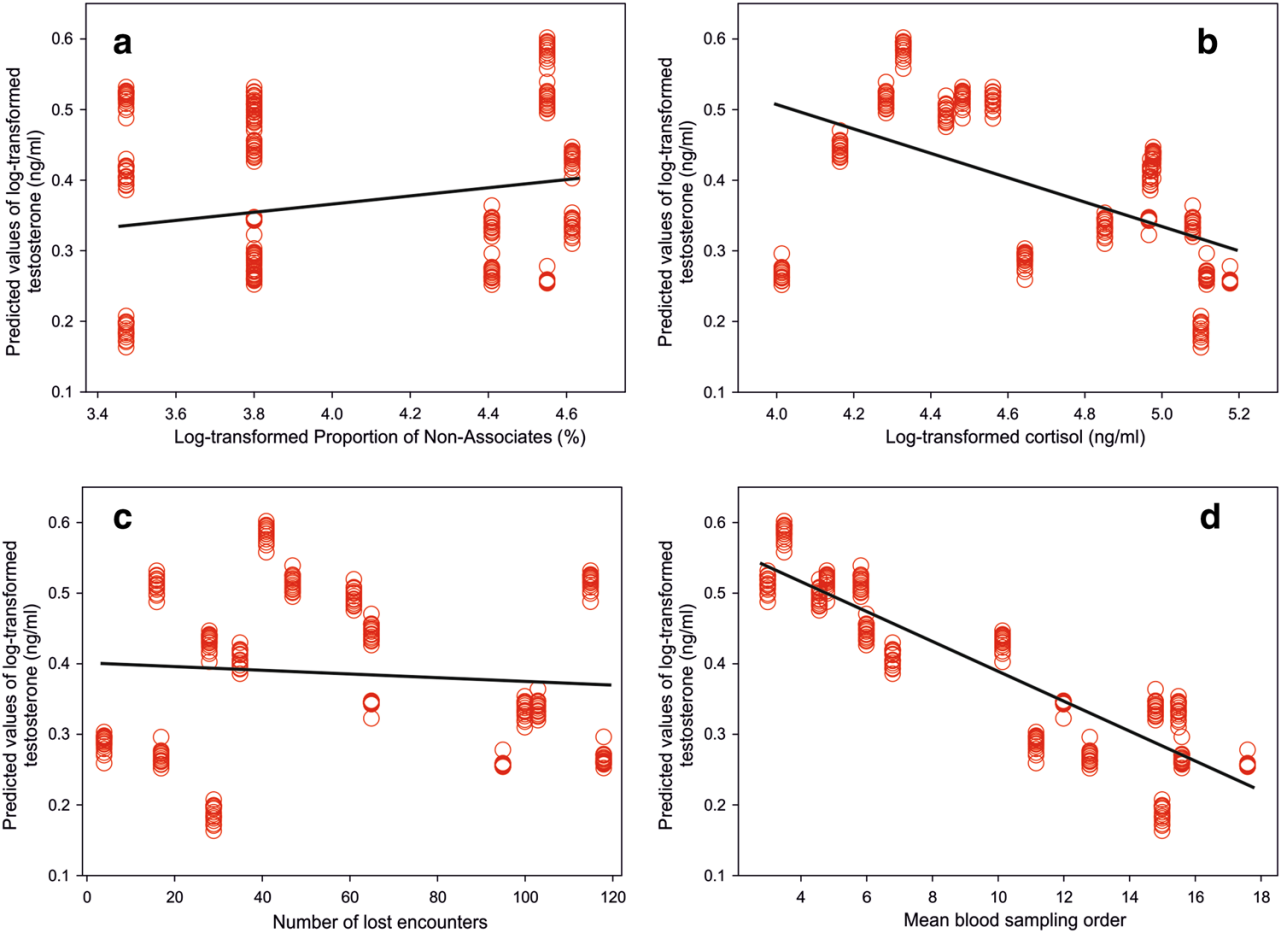
Predicted values of Testosterone concentrations (ng/ml) plotted against log-transformed Proportion of Non-Associates (%) (**a**), log-transformed concentrations of Cortisol (ng/ml) (**b**), Number of lost encounters (**c**), and Mean blood sampling order (**d**).

Prediction (iii) Five GLMMs for log-transformed T concentrations were uniformly ranked by all criteria of best fit (Supplementary Table S1). A comparison of the models fitted showed that the best model has merit with zero relative information loss estimated by all five fit criteria (Supplementary Table S2). The best model was sufficiently distant from the second-best model (Table 3, Δ = 4.49 was the same for all fit criteria), with the correct model’s probability of 84% (the second-best model 0.09%). The best model was more than 9.44 times (odds) more likely to be the correct model than the second one. The best model consisted of four fixed effects; Proportion of Non-Associates (Fig. 2a, T concentrations increased with increasing proportion of Non-Associates), log-transformed C concentrations (Fig. 2b, T concentrations decreased with the increasing C concentrations), number of lost encounters (Fig. 2c, the T concentrations decreased with the decreasing number of lost encounters), and mean blood sampling order (Fig. 2d, as the blood sample was taken later, the T concentrations became lower). Estimates, Standard error and 95% confidence interval for best fitting GLMM model for T are shown in the Supplementary Table S3.

Prediction (iv) In contrast with our expectation, the effect of Dominance was not observed in any of the top fitting GLMMS, neither for C concentrations nor T concentrations.

**Figure 3:**
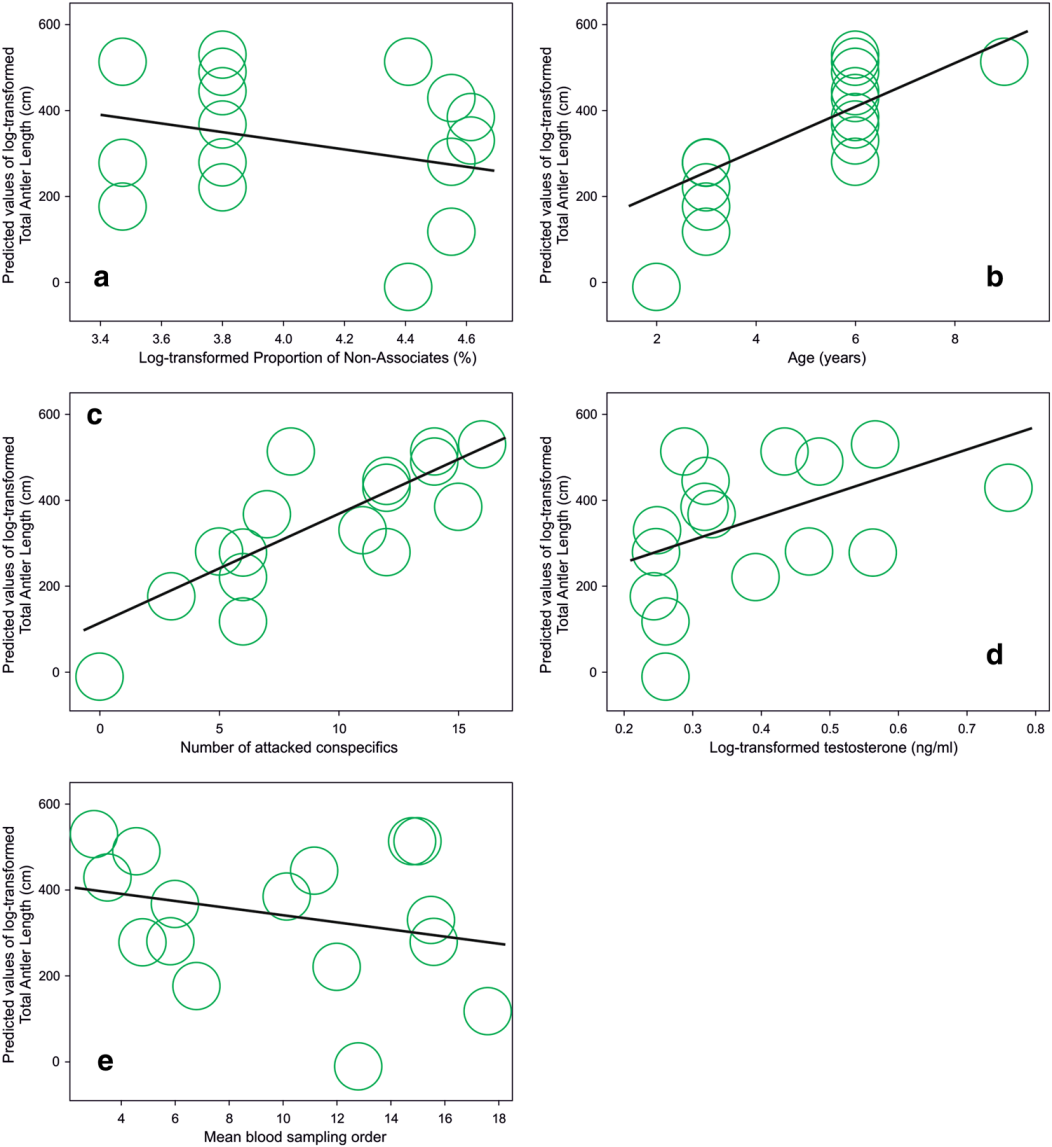
Bubble graphs showing predicted values of Total antler length (cm) plotted against log-transformed Proportion of Non-Associates (%) (**a**), Age (years) (**b**), Number of attacked conspecifics (**c**), log-transformed Testosterone concentrations (ng/ml) (**d**), and Mean blood sampling order (**e**). (Each bubble represents 16 values in the same position).

Total antler length was explained by the highest number of potential fixed factors. All five fit criteria nominated and ranked the same best 5 models (Supplementary Table S1). Again, in full agreement across all fit criteria (*Δ* = 6.21), the best model was convincingly the best (Supplementary Table S2, Table 3), with the high correct model's probability (0.96%) in comparison to the second model (0.04%), and high odds (22.27). The best model revealed that total antler length tended to decrease with the increasing proportion of Non-Associates (Fig. 3a). On the other hand, the total antler length increased as the males aged (Fig. 3b), with the increasing number of males attacked (Fig. 3c), with the rising T concentrations (Fig. 3d). Increasing the mean blood sampling order decreased T concentrations (Fig. 3e). The Supplementary Table S3 shows estimates, standard error and 95% confidence interval for best fitting GLMM model for this dependent variable.

In order to eliminate results which are simply due to internal characteristics of individual males rather than the result of short-term social strategy, the association of the hormonal and antler measures of the same individual males between Season 1 and Season 2 and between Season 2 and Season 3 was estimated by Kendall’s and Lin’s concordance correlation coefficients (Table 4***)***. Lin’s Concordance Correlation and Kendall’s correlation coefficients comparing Season 1 and Season 2 were low for C and T concentrations, much lower when comparing the concordance between Season 1 and Season 2 than those between Season 2 and Season 3. On the contrary, the correlations for Total antler length were high and did not differ between Season 1—Season 2 and Season 2—Season 3.

**Table 4:** Estimate of Kendall’s and Lin’s concordance correlation coefficients for the association of the hormonal and antler measures of the same subjects between Season 1 and Season 2 and between Season 2 and Season 3.

Seasons compared	N	Mean 1	Mean 2	Variance 1	Variance 2	Covariance	Corr Lower CL	Kendall ‘s Corr. Coeff. τ Probab	Corr upper CL	Conc. corr. lower CL	Conc. corr ρ_c_	Conc. corr. upper CL
Cortisol												
Season1–Season2	13	105.58	93.26	1450.38	1282.13	144.70	− 0.18	0.18 *P* = 0.87	0.49	− 0.48	0.100	0.62
Season2–Season3	12	95.16	91.92	1347.53	1363.15	519.10	0.02	0.42 *P* = 0.04	0.71	− 0.29	0.382	0.80
Testosterone												
Season1–Season2	13	0.41	22.28	0.05	223.37	1.30	− 0.15	0.21 *P* = 0.26	0.51	− 0.003	0.004	0.01
Season2–Season3	12	22.80	33.56	239.90	878.29	222.59	− 0.05	0.36 *P* = 0.08	0.67	− 0.13	0.361	0.71
Total antler length												
Season1–Season2	13	345.38	454.35	23,090.63	23,684.39	20,263.04	0.31	0.59 *P* < 0.001	0.78	0.32	0.691	0.88
Season2–Season3	12	475.08	488.51	19,738.90	6506.39	9103.97	0.14	0.52 *P* < 0.01	0.76	0.33	0.689	0.87

## Discussion

As far as we know, this is the first study documenting alternative social strategies applied within the same group of a gregarious species, with individuals apparently either seeking the benefit of the “social buffering” or the stimulating effect of the “challenge hypothesis”. Our study has shown that social buffering or challenging could have an essential impact on C and T concentrations; for this, individual males were highly selective in joining or avoiding the company of others. This was in turn, reflected in the variability in antler growth.

As expected (i), a proportion of the males preferred company of some individuals for a prolonged time, but not of others. The greater inter-individual distance did not prevent agonistic encounters with some “Distant” (i. e. non-associated) deer. However, some pairs of males consistently avoided each other even when aggregated during supplemental feeding. The reason why some males tended to keep in proximity with others or not could have been based on their personality^49^, inter-individual relationships and previous experience^50^, laterality of brain function and/or actual physical condition^51^.

The second prediction (ii) was also fulfilled. Potentially stressful situations occurred throughout the whole period of observation. Close social partners apparently were able to buffer against the adverse effects of increased physiological stress levels induced by the attacks from others^30^. Indeed, with increasing proportion of Associates in the interactions, C concentrations decreased (Fig. 1a). Thus, this study suggests a calming effect of social bonding among the red deer males under the conditions where the male might decide whether to join the proximity of tolerant, sociable conspecifics or others. Aggregating with others in a socially stable company was thus apparently a tactic which offers benefits through the “social buffering” effect^28^. Supporting the idea of a general effect of the “social buffering hypothesis”^28^ beyond the most frequently described mother–offspring, kin or pair bond^30,33,52,53^. It also offers evidence for buffering effects extending to a wider range of species, while previous reports have been restricted to humans^35^ and phylogenetically closely related primates^33^. The results of this study may also support the suspicions of Hennessy, et al.^30^ according to whom there are many cases in which ”social buffering “ of the HPA axis exists but has not been documented. This increases the importance of the current results because they provide evidence under controlled conditions while maintaining the free will of the individual males on which strategy to choose.

The fact that increasing body weight was associated with an increase of C concentration may reflect an increasing energy demand of larger individuals, as previously shown in the red deer^15,49^. On the other hand, the increasing T concentrations associated with decreasing C concentrations are difficult to explain. In general, increasing T concentrations should also elicit increasing C concentrations to mobilize energy^23^. Although the C concentrations were the dependent variable, the low T concentrations associated with high C concentrations could have been a side effect of a general blocking effect of C over T concentrations, as documented in many previous studies^15,19,21^.

(iii) The males had known each other for an extended period before observations of the present study began. However, interactions reflecting an avoidance of socially stable groupings and attacking Non-associates increased T concentrations (Fig. 1d). This strongly supported an effect of a challenging encounter, as anticipated^16,46,47^. In another study in the same herd, we have found that even though individual males had shown some plasticity in their behavioural response, the style of their individual inclination towards interaction had been maintained over three consecutive years despite the experimental modifications of the social environment^49^. Thus: those who tended to be aggressive against others were aggressive in any social situation, while those tending to avoid conflict also maintained this strategy in any social conditions. This would fit the opinion of Bell^54^ who has suggested that when individuals have a behavioural type that is stable over time or across situations, this could cause “behavioural spillovers” and limited plasticity. Despite this, the relations between challenging situations and T elevation were pronounced.

Indifferent males did not interact and therefore did not apply any of the two tactics.

The current study did not show a real suppressive effect of C concentrations on T concentrations, which would suggest that competition between males under the particular social conditions was resulting in stress^19,21^. However, the lack of evidence of the suppressive effect of C concentrations on T concentrations may reflect the relatively stable social situation.

(iv) There was no visible effect of dominance on T concentrations as such. The effect of dominance was not in the models of best fit for either C or T concentrations nor for total antler length. Still, it was among the best five GLMMs for the dependent variables C and T concentrations (Table S1). The positive effect of the increasing total of attacked conspecifics on antler growth (Fig. 3c) also suggested involvement of an effect of dominance throughout increased aggression of the males. Such a role of the “invisible”, but nonetheless present dominance is in agreement with our previous studies^3,55^. Details obtained in the current study showed that association with tolerant individuals or attacking Non-Associates was a more potent factor than dominance, as we previously thought^10,55^.

Antler growth in this study revealed dependency on age, overall frequency of attacks (number of attacked conspecifics) and T concentrations, in full harmony with previous studies^4,5^. The association with tolerant, sociable conspecifics, thus enjoying the effect of “social buffering”^28^, does not seem sufficient to explain the production of larger antlers despite reducing C concentrations (Fig. 1a). Therefore, under the spatially restricted conditions of our study, a male deer tending to avoid regular association with others tended to develop the largest antlers. It probably benefited mainly from frequent victorious agonistic threats to conspecifics with mutually less stable dominance relationships. In accordance with the “challenge hypothesis”^45^, attacking such conspecifics most likely led to the elevation of T concentrations^16,44,46^ necessary for antler growth^4^. Besides that, our results suggest a long-lasting effect of repeated challenging encounters.

The concordance and Kendall’s correlation coefficients of hormonal values between Season 1 and Season 2 were generally very low. Moreover, they were lower than those between Season 2 and Season 3, when the males lived in the same environmental conditions. The low correlations and the difference between Season 1- Season 2 and Season 2- Season 3 strongly support the presumption that both C and T concentrations depended on the males’ tactics rather than some other individual characteristic. It also supports the conclusion that the choice between actively seeking or avoiding association is effective regarding social buffering and challenge hypothesis principles. Relatively high correlation coefficients of the total antler length between Season 1- Season 2 and Season 2- Season 3 correspond to high repeatability of antler size previously reported^56^. The high repeatability of the total antler length may be based on the general tendency of phenotypic persistence of the antler shape e.g.,^57^. That is why although we have shown an effect of seeking or avoiding association on C and T concentrations (Fig. 1) and apparent dependency of the total antler length on resulting T concentrations (Fig. 2), modification of the antlers did not change their primary shape that much.

Besides other results, our current study emphasised the necessity to consider the time that had elapsed since people started handling the animals to the time of the blood sample collection. This factor affected all dependent variables, i. e., hormone concentrations and the total antler length.

## Conclusions

In conclusion, association with others appeared a potent factor affecting all three dependent variables. Interacting with a higher proportion of Associates was associated with lower C concentrations. Indirectly, it affected T concentrations in a way that the proportion of Non-Associates, an almost mirror opposite variable to the proportion of Associates, associated with the increased T secretion. The suggested trade off situation that combines the “social buffering hypothesis”^28,30,33^ and “challenge hypothesis” effect^16,45,48,58^ should be further investigated in less spatially constrained situation or in free living populations. Still, our study animals, the red deer, can be taken as a model species with the possibility to be applied of applying this methodology to other social animals.

## Methods

Observation of farmed red deer took place in a deer facility belonging to the Institute of Animal Science (V.Ú.Ž.V.) at Podlesek, Praha, Czech Republic (50°03′02.2"N 14°35′37.1"E).

Setting up a classical control is a problem in a study like this. Without it, we could not fully distinguish whether the social environment causes differences in hormone levels and antler growth or whether specific individuals (with particular hormonal levels and antler growth characteristics) are more or less likely to aggregate with others. Without a full control, it is not possible to discount the hypothesis that internal characteristics of individual males could influence our results. We have previously shown that antler size has high repeatability in subsequent seasons during ontogeny^56^. Moreover, even though the males had shown behavioural plasticity, their individual attitude to seeking or avoiding interaction had been maintained despite the modifications of the social environment^49^.

In addition to our main experimental season (Season 1), we also had data on hormones and antler sizes from the following two seasons (Season 2 and Season 3). We, therefore, presumed that if the cause of the results were based on characteristics which were individual-specific, then the hormonal and antler values recorded for individual males in this study in Season 1 should correlate with those of the same individuals in the following season or seasons. On the other hand, if the hormonal and antler characteristics displayed by individual males in Season 1 were primarily a consequence of the males’ social tactics, the repeatability of hormonal and antler values between Season 1 and Season 2 should be lower than that between Season 2 and Season 3, when the males lived in the same environmental conditions.

### Study animals

Seventeen semi-tame red deer males (one male aged 9, seven aged 6, six aged 3, and three aged 2) belonging to the same bachelor group since birth were available at the beginning of the observation at the facility, within an area of approximately 4 ha. This area was divided into six enclosures. Each enclosure (about 0.7 ha large) contained a shelter (a wooden, roofed barn with one side permanently open with the entrance of approximately 24 m^2^), a water reservoir, and a mud pool for wallowing. During the main observation period (from 17^th^ April to 28^th^ August in Season 1, the period of antler growth), all enclosures were interconnected by two (in the first and last enclosures) or three permanently opened gates in other enclosures allowing the deer to move around and aggregate with or separate from others. In contrast, for “control” purposes, in Seasons 2 and 3, the same males were kept in three interconnected enclosures, each 0. 7 ha in size, i. e. their living space in Seasons 2 and 3 represented about 50% of the area in Season 1. The animals fed predominantly on the natural pasture of the enclosures and were supplemented with hay (ad libitum) and occasionally also with potatoes, beets, apples, pears, barley and oats. The animals were identified with coloured, numbered collars and in Season 1 equipped also with GPS collars (Lotek Wireless Inc. GPS_3300, position readings with an error of less than 5 m). When this study ceased, all animals stayed at the facility for future investigations.

### Animal welfare

According to European and Czech laws, the experimental deer facility is an accredited research centre for the ethical use of animals in research (60444/2011- MZE-17214). The experimental proposal no. MZe 1297 was approved by the Animal Care and Use Committee at the Ministry of Agriculture of the Czech Republic. We confirm that all methods were carried out following relevant guidelines and regulations and are reported in accordance with ARRIVE guidelines.

### Data collection

In Season 1, observations were designed to record agonistic interactions between animals when competing for supplemental food. At the time of observation, deer were fed a mixture of soya, barley, oats and a mineral/vitamin premix, which amounted to an average of 0.7 kg/day/animal. When the supplemental food was presented, it usually attracted all males regardless of whether they were otherwise in groups or associated individually in the paddocks. Thus, at the time of provision of the supplementary food, all the males met together even if otherwise they preferred to avoid encounters with other individuals.

The food was carried to the observation place in a wheelbarrow and presented in several piles to encourage mild competition over a scarce resource (serious competition was prevented to preserve good welfare of the stags). The food piles were tipped from the wheelbarrow in 8 or 9 piles about 2 m apart, in order to encourage competition without exacerbating it. This method has already been proven valid in previous studies e. g.,^15,49^, etc. Each observation session took place in the morning (between 9.00 a.m. and 11.30 a.m.) and ranged from 20 to 60 min (depending how long the deer stayed at the site of supplementary feeding). In Seasons 1, 2, and 3, observations took place from 1 to 5 times per week between 1st May and 28th August (with an average equal to 3). In total, the deer interactions were observed for 37 h in Season 1, 30 h in Season 2, and 15 h in Season 3. All deer were semi-tame and started to compete over the food as soon as it had been presented, running from one pile to another trying to eat as much as possible. When a feeding deer was challenged by others, it either escaped to another pile or defended itself. All the observations were made into a voice recorder and then transcribed into a table using Microsoft Excel. We recorded any occurrence of an approach of one male to another, any attack, threat gesture, or fighting, which caused an apparent displacement of the approached individual^49,59^. As in previous studies reviewed in^55^, we determined dominance status for each pair of males on the basis of the agonistic interactions observed. We regarded as “dominant” the males who won more agonistic encounters than they lost in any dyad, as “subordinate” the ones who lost more often than they won within the dyad, and as “indifferent” the males in a dyad with no agonistic interactions.

GPS collars measured inter-individual distances between males in Season 1 only. Positions were programmed to be recorded once per hour. This enabled us to obtain records of inter-individual distances during the observation period with an average of 90.40 ± 4.6 m (mean ± SE) per dyad (n = 272) over the observation period, producing a reliable picture of mean inter-individual spaces whole period. In Seasons 2 and 3, we did not use GPS collars and made no detailed spatial observations as done in Season 1.

In all three study years, we weighed the males once a month (5 times between April and August), collected blood samples for the hormone analysis in a physical restraining facility (“crush”). All deer involved were used to this procedure and had undergone it since birth. No chemical restraint was used besides physical restraint. In Season 1, when collecting blood samples, we downloaded GPS records from data loggers. In all seasons, we measured the antlers after casting, as previously described e. g.,^60^ and used the total antler length, the final sum of the length of all tines, points and beams divided by 2^61^, as a dependent variable.

### Hormone analyses

Analyses of T and C concentrations were performed in the laboratories of ELISA development, s.r.o. (Velké Žernoseky, the Czech Republic). T concentration was measured by RIA Kit from Beckam Coulter (code IM1087). T antibody for this RIA Kit is species-nonspecific. The radioimmunoassay of T is a competitive assay. Before the assay, plasma samples were extracted with ethyl ether; the solvent was evaporated, and the dry residues were re-dissolved in the recovery buffer of the kit. The re-dissolved extracts and calibrators were then incubated with ^125^I-labeled T, as a tracer, in an antibody-coated tube. The concentration range was up to 23 ng/mL, the assay's detection limit was 0.1 ng/mL, intra-assay-precision was 8.6%, and inter-assay was 11.9%. The recovery of the extraction step was 90%.

C concentration was determined by RIA Kit from Beckman Coulter (code IM1841) previously validated only in cattle^62,63^. C antibody for this RIA Kit is also species-nonspecific. The radioimmunoassay of C is a competitive assay. Samples and calibrators were incubated in monoclonal antibody-coated tubes with ^125^I-labeled cortisol tracer. The concentration range was up to 2000 nM, the assay's detection limit was 5 nM, intra-assay-precision was 9.4%, and inter-assay was 12.6%.

### Statistics

All data were analysed with the aid of the SAS System (SAS, version 9.4).

Previous studies have shown that it is essential in assessing relationships between social behaviour and physiology to record and analyse measured characteristics in as much detail as possible e. g.,^6^. Therefore, we preferred to analyse the inter-individual pairwise relationships rather than rely upon any form of summarized values.

(i) In the main observation period (Season 1), for each male, we collected for each observation the inter-individual mean distance (meters) from each of the herd mates (i.e., 16 inter-individual distances per male). A cluster analysis (PROC CLUSTER, with TYPE = NOMINAL and METHOD = HIERARCHICAL) was used to divide the mean inter-individual distances resulting into two groups, “Associates” (males keeping together) and “Distant” (those living apart). According to their involvement in interactions during the feeding competition, these latter (distant) dyads were further subdivided as “Indifferent” (i.e., no interaction within the dyad was recorded), or „Non-associates “ (i. e., dyads keeping mutual distance, interacting during the feeding competition only). In conclusion, three levels of mutual relationship between the individual stags were considered: “Associates” (keeping together), “Non-associates” (keeping distance but interacting when meeting during the feeding) and “Indifferent” (keeping distance, non-interacting). For each male, we then calculated the "Proportion of Associates" of all dyadic relationships (% of individuals from all 16 possible dyadic groups who were identified as Associates of the focal individual) and the Proportion of Non-Associates relationships (% of individuals identified as Non-Associates within any dyadic group). At this point, however, it should be pointed out that the classification of Associates, Non-Associates and Indifferent concerns dyadic distances, not the categorization of males. Thus, each individual could be Associate with one male, Non-Associate with another male, and Indifferent with other males. It, therefore, depended on whom the focal individual had interactions with, and which conspecifics preferred more than others.

For each subject we had available also other characteristics of interactions (listed in Table 2), between him and all other males such as the number of attacks, wins, losses, etc. For the analysis, we used the mean values of all quantifiable variables over the whole period for each subject and all its dyads. Having 17 males with 16 relationships each, we obtained 272 dyadic records in total.

To check for possible multicollinearity, we first calculated correlations (PROC CORR) between the individual metrics involved (Table 2). Significant correlation was found between the Bodyweight at the beginning of the observation and at the end of the entire experimental period (May–August; r = 0.91, *P* < 0.0001), between Bodyweight and Weight gain (r = 0.83, *P* < 0.0001), between Age and Bodyweight (at the beginning of the observation r = 0.84, *P* < 0.0001; and at the end of the observation r = 0.71, *P* < 0.0001. We subsequently made a judgment of the extent of collinearity by checking related statistics, such as Tolerance value, Variance Inflation Factor (VIF), Eigenvalue, and Condition Number and using TOL, VIF and COLLIN options of the MODEL statement in the SAS REG procedure. We discovered apparent collinearity between all variables characterizing agonistic interactions (i. e., Sum of all agonistic interactions of any given type, Wins, Losses, and Number of attacked conspecifics). When either of these characteristics entered the REG procedure alone, the lowest tolerance value did not drop below 0.13. The highest variance inflation value did not exceed the value of 7.5. Also, there was no case of small eigenvalues combined with the large corresponding condition number. So, there was no threat of other multicollinearity indicated by these results.

Across the models, where appropriate, count variables were log-transformed (natural logarithm transformation) to improve the normality of residuals and to reduce skewness.

Since the issues analysed in this study represent more complex causality, we used the information-theoretic approach (IT-AIC) for estimating the effects of the factors on dependent variables^64^.

Associations were subsequently sought between C concentrations (ii), T concentrations (iii), or total antler length as dependent variables and the remaining fixed factors (Table 2) using a multivariate General Linear Mixed Model (GLMM, PROC MIXED). To account for the repeated measures on the same individuals, all analyses were performed using PROC MIXED with ID of the individual male as a random effect. For each dependent variable, we constructed a set of multiple a priori hypotheses and added a Null model. Where appropriate, we included interaction terms. Specifically, for log-transformed C concentrations, we set up 38 alternative hypotheses, for log-transformed T concentrations 26 hypotheses, and for Total antler length 90 hypotheses (Supplementary Table S4). For each dependent variable (i.e., C, T, and total antler length) we generated all GLMMs in the Supplementary Table S4 and converted values of fit statistics.

Since the introduction of Akaike's Information Criterion (AIC), more information criteria have been developed with differing mathematical properties and philosophies of model selection^65^. We used expanded information criteria AIC, AICC, BIC, CAIC, and HQIC to select a true model, as recommended by Christensen^65^. Then we compared the candidate models by ranking them based on the information criteria being used (PROC RANK). The model with the lowest value (i. e. closest to zero) is considered to be the "best" model^64,65^. To see if the best model has merit, we compared our model to the null model for all dependent variables and all fitting criteria, showing delta (null – best model) and a relative information loss [exp((null − best)/2)], an approach adapted from Burnham and Anderson^64^.

The differences (Δ_*i*_) between the Fit statistic values (the smallest values indicating the best fitting model) were sorted according to AIC values. Akaike weight *w*_*i*_ can be interpreted as the probability that M_*i*_ is the best model (in the AIC sense, that it minimizes the Kullback–Leibler discrepancy), given the data and the set of candidate models e. g.,^64^. For five models with the lowest AIC values, we therefore calculated Δ AIC, Akaike weights *wi*, and for estimating the strength of evidence in favour of one model over the other we divided their Akaike weights *w*_*min*_*/w*_*j*_ (AIC Odds)^64^.

Associations between the dependent variable and countable fixed effects are presented by fitting a random coefficient model using GLMM as described by Tao et al.^66^. We calculated predicted values of the dependent variable and plotted them against the fixed effects with predicted regression lines.

Several statistical methods are typically used to show “comparability” or “repeatability^67^”. As previously^49^, we chose Lin’s concordance correlation coefficient^68^ using the SAS macro described by^67^ and Kendall’s tau-b correlation coefficient to estimate a measure of association of the hormonal and antler measures of the same subjects between Season 1 – Season 2, and between Season 2 – Season 3. For computing Kendall’s correlation coefficients and its confidence interval estimation, we applied macro by Looney^69^. From the Seasons 1 to 3, males group consisted of the same individuals. Decreasing N on comparisons between seasons (Table 4) reflected that some males were removed from the facility for other purposes (four in Season 2 and one in Season 3).

### Supplementary Information

Supplementary Information.

## Data Availability

If a reader needs data used in this study, the authors are ready to supply the data under a formal request with suitable reasons. Correspondence and requests for materials should be addressed to L. B.
