# Supplementary material for: The effects of challenge or social buffering on cortisol, testosterone, and antler growth in captive red deer (Cervus elaphus) males
Source: Sci Rep. 2023 Dec 9;13:21856. doi: 10.1038/s41598-023-48476-9 (PMC10710442; doi:10.1038/s41598-023-48476-9)
Supplement: Supplementary file 1 — Supplementary Information. [file 41598_2023_48476_MOESM1_ESM.pdf]

The effects of challenge or social buffering on cortisol, testosterone, and antler growth in captive red deer (*Cervus elaphus*) males

Luděk Bartoš, Bruno Esattore, Radim Kotrba, Jan Pluháček, Francisco Ceacero, Martina Komárková, Adam Dušek, Jitka Bartošová

Table S1. Five best candidate models and Null model with values of the expanded information criteria sorted by AIC (from the lowest to the highest value) and ranked (the number in parentheses correspond to a rank within the criterion). (The letter "t" at the end of the term means "log-transformed".)

Log-transformed cortisol					
Model	AIC	AICC	BIC	CAIC	HQIC
Proc_Asst Testosteronet Weight2t Order	25.32 (1)	25.37 (1)	21.32 (1)	23.32 (1)	21.32 (1)
Proc_Asst Testosteronet Weight2t Dominance Order	34.28 (2)	34.33 (2)	30.28 (2)	32.28 (2)	30.28 (2)
Proc_Asst Testosteronet Number_attacked Order	54.81 (3)	54.86 (3)	50.81 (3)	52.81 (3)	50.81 (3)
Proc_Asst Testosteronet Number_attacked Dominance Order	63.31 (4)	63.36 (4)	59.31 (4)	61.31 (4)	59.31 (4)
Proc_Asst Testosteronet Tot_INTERACT Order	68.86 (5)	68.91 (5)	64.86 (5)	66.86 (5)	64.86 (5)
Null Model	221.13 (6)	221.14 (6)	224.67 (6)	225.67 (6)	222.55 (6)
Log-transformed testosterone					
Model	AIC	AICC	BIC	CAIC	HQIC
Proc_NAsst cortisolt Losses Order	-461.47 (1)	-461.42 (1)	-465.47 (1)	-463.47 (1)	-465.47 (1)
Proc_NAsst cortisolt Tot_INTERACT Order	-456.98 (2)	-456.93 (2)	-460.98 (2)	-458.98 (2)	-460.98 (2)
Proc_NAsst cortisolt Losses Dominance Order	-455.48 (3)	-455.43 (3)	-459.48 (3)	-457.48 (3)	-459.48 (3)
Proc_NAsst cortisolt Age Order	-453.45 (4)	-453.40 (4)	-457.45 (4)	-455.45 (4)	-457.45 (4)
Proc_NAsst cortisolt Order	-452.89 (5)	-452.84 (5)	-456.89 (5)	-454.89 (5)	-456.89 (5)
Null Model	-253.28 (6)	-253.26 (6)	-249.74 (6)	-248.74 (6)	-251.85 (6)
Total antler length					
Model	AIC	AICC	BIC	CAIC	HQIC
Proc_NAsst Age Number_attacked Testosteronet Order	2793.27 (1)	2793.32 (1)	2789.27 (1)	2791.27 (1)	2789.27 (1)
Proc_NAsst Age Number_attacked Testosteronet	2799.48 (2)	2799.53 (2)	2795.48 (2)	2797.48 (2)	2795.48 (2)

Proc_NAsst Age Number_attacked Cortisolt Order	2817.00 (3)	2817.02 (3)	2815.00 (3)	2816.00 (3)	2815.00 (3)
Proc_NAsst Age Number_attacked Cortisolt	2825.37 (4)	2825.38 (4)	2823.37 (4)	2824.37 (4)	2823.37 (4)
Proc_Asst Age Number_attacked Cortisolt Order	2825.83 (5)	2825.84 (5)	2823.83 (5)	2824.83 (5)	2823.83 (5)
Null Model	3327.07 (6)	3327.08 (6)	3330.61 (6)	3331.61 (6)	3328.49 (6)

Table S2. Comparison of the best model to the null model, delta (Δ), and a relative information loss for log-transformed cortisol, log-transformed testosterone, and total antler length according to five fit criteria.

Log-transformed cortisol				
Criterion	Null model	Best model	Δ	Relative information loss
AIC	225.86	116.44	109.42	1.73418E-24
AIC	221.13	25.32	195.81	3.02554E-43
AICC	221.14	25.37	195.78	3.07522E-43
BIC	224.67	21.32	203.35	6.97009E-45
CAIC	225.67	23.32	202.35	1.14917E-44
HQIC	222.55	21.32	201.23	2.00863E-44
Log-transformed testosterone				
Criterion	Null model	Best model	Δ	Relative information loss
AIC	-253.28	-461.47	208.19	6.19727E-46
AICC	-253.26	-461.42	208.16	6.30E-46
BIC	-249.74	-465.47	215.73	1.4277E-47
CAIC	-248.74	-463.47	214.73	2.35387E-47
HQIC	-251.85	-465.47	213.61	4.11431E-47
Total antler length				
Criterion	Null model	Best model	Δ	Relative information loss
AIC	3327.07	2793.27	533.79	1.2248E-116
AICC	3327.08	2793.32	533.76	1.245E-116
BIC	3330.61	2789.27	541.34	2.8217E-118
CAIC	3331.61	2791.27	540.34	4.6521E-118
HQIC	3328.49	2789.27	539.22	8.1314E-118

Table S3. Estimate, Standard error and 95% confidence interval for best fitting GLMM models for dependent variables log-transformed cortisol, log-transformed testosterone and total antler length ("t" at the end of the effect's name in a model means "log-transformed")

For the dependent variable cortisolt				
Effect	Estimate	StdErr	Lower	Upper
Intercept	2.10	0.22	1.66	2.54
Proc_Asst	0.03	0.01	0.01	0.05
Testosteronet	-0.18	0.16	-0.48	0.13
Weight2t	0.41	0.04	0.32	0.49
Order	0.05	0.00	0.04	0.06
For the dependent variable testosteronet				
Effect	Estimate	StdErr	Lower	Upper
Intercept	-0.03	0.12	-0.27	0.20
Proc_NAsst	0.10	0.01	0.07	0.13
Cortisolt	0.05	0.02	0.00	0.09
Losses	0.00	0.00	0.00	0.00
Order	-0.03	0.00	-0.03	-0.02
For the total antler length				
Effect	Estimate	StdErr	Lower	Upper
Intercept	321.27	37.65	247.13	395.41
Proc_NAsst	-119.39	9.96	-139.00	-99.78
Age	53.76	2.60	48.64	58.88
Number_attacked	13.30	1.01	11.31	15.30
Testosteronet	209.78	40.68	129.68	289.88
Order	2.46	1.22	0.07	4.86

Table S4. A set of multiple a priori hypotheses for the dependent variables Cortisol and Testosterone concentrations, and Total antler length ("t" in the end of the name of the variable means "log-transformed")

Log-transformed Cortisol	
No.	Models
1	Proc_Ass
2	Proc_Asst Dominance
3	Proc_Asst Testosteronet
4	Proc_Asst Testosteronet Age
5	Proc_Asst Testosteronet Age Dominance
6	Proc_Asst Testosteronet Aget
7	Proc_Asst Testosteronet Aget Dominance
8	Proc_Asst Testosteronet Dominance
9	Proc_Asst Testosteronet Losses
10	Proc_Asst Testosteronet Losses Dominance
11	Proc_Asst Testosteronet Number_attacked
12	Proc_Asst Testosteronet Number_attacked Dominance
13	Proc_Asst Testosteronet Tot_INTERACT
14	Proc_Asst Testosteronet Tot_INTERACT Dominance
15	Proc_Asst Testosteronet Weight2
16	Proc_Asst Testosteronet Weight2 Dominance
17	Proc_Asst Testosteronet Weight2t
18	Proc_Asst Testosteronet Weight2t Dominance
19	Proc_Asst Testosteronet Wins
20	Proc_Asst Testosteronet Wins Dominance
21	Proc_Asst Testosteronet Order
22	Proc_Asst Testosteronet Age Order
23	Proc_Asst Testosteronet Age Dominance Order
24	Proc_Asst Testosteronet Aget Order
25	Proc_Asst Testosteronet Aget Dominance Order
26	Proc_Asst Testosteronet Dominance Order
27	Proc_Asst Testosteronet Losses Order
28	Proc_Asst Testosteronet Losses Dominance Order
29	Proc_Asst Testosteronet Number_attacked Order
30	Proc_Asst Testosteronet Number_attacked Dominance Order
31	Proc_Asst Testosteronet Tot_INTERACT Order
32	Proc_Asst Testosteronet Tot_INTERACT Dominance Order
33	Proc_Asst Testosteronet Weight2 Order
34	Proc_Asst Testosteronet Weight2 Dominance Order
35	Proc_Asst Testosteronet Weight2t Order

36	Proc_Asst Testosteronet Weight2t Dominance Order
37	Proc_Asst Testosteronet Wins Order
38	Proc_Asst Testosteronet Wins Dominance Order
39	Null
Log-trasformed Testosterone	
1	Proc_NAsst
2	Proc_NAsst Dominance
3	Proc_NAsst cortisolt
4	Proc_NAsst cortisolt Age
5	Proc_NAsst cortisolt Age Dominance
6	Proc_NAsst cortisolt Dominance
7	Proc_NAsst cortisolt Losses
8	Proc_NAsst cortisolt Losses Dominance
9	Proc_NAsst cortisolt Number_attacked Dominance
10	Proc_NAsst cortisolt Tot_INTERACT
11	Proc_NAsst cortisolt Tot_INTERACT Dominance
12	Proc_NAsst cortisolt Weight2
13	Proc_NAsst cortisolt Wins
14	Proc_NAsst cortisolt Wins Dominance
15	Proc_NAsst cortisolt Order
16	Proc_NAsst cortisolt Age Order
17	Proc_NAsst cortisolt Age Dominance Order
18	Proc_NAsst cortisolt Dominance Order
19	Proc_NAsst cortisolt Losses Order
20	Proc_NAsst cortisolt Losses Dominance Order
21	Proc_NAsst cortisolt Number_attacked Dominance Order
22	Proc_NAsst cortisolt Tot_INTERACT Order
23	Proc_NAsst cortisolt Tot_INTERACT Dominance Order
24	Proc_NAsst cortisolt Weight2 Order
25	Proc_NAsst cortisolt Wins Order
26	Proc_NAsst cortisolt Wins Dominance Order
27	Null
Total Antler length	
1	Age
2	Aget
3	Weight2t
4	Dominance
5	Proc_Asst Age Cortisolt Testosteronet
6	Proc_Asst Age Number_attacked
7	Proc_Asst Age Number_attacked Cortisolt
8	Proc_Asst Age Number_attacked Testosteronet
9	Proc_Asst Age Tot_INTERACT
10	Proc_Asst Age Tot_INTERACT Cortisolt

11	Proc_Asst Age Tot_INTERACT Testosteronet
12	Proc_Asst Age Wins
13	Proc_Asst Age Wins Cortisolt
14	Proc_Asst Age Wins Testosteronet
15	Proc_Asst Losses Weight2
16	Proc_Asst Weight2 Wins
17	Proc_Asst Weight2 Wins Cortisolt
18	Proc_Asst Weight2 Losses
19	Proc_Asst Weight2 Tot_INTERACT
20	Proc_Asst Weight2 Tot_INTERACT Cortisolt
21	Proc_Asst Weight2 Tot_INTERACT Testosteronet
22	Proc_Asst Weight2 Wins
23	Proc_Asst Weight2 Wins Cortisolt
24	Proc_Asst Weight2 Wins Testosteronet
25	Proc_Asst Wins
26	Proc_Asst Wins Cortisolt
27	Proc_Asst Wins Testosteronet
28	Proc_Asst Wins Weight2
29	Proc_Asst Wins Weight2 Cortisolt
30	Proc_Asst Wins Weight2 Testosteronet
31	Proc_Asst Age Cortisolt Testosteronet Order
32	Proc_Asst Age Cortisolt*Testosteronet Order
33	Proc_Asst Age Number_attacked Cortisolt Order
34	Proc_Asst Age Number_attacked Testosteronet Order
35	Proc_Asst Age Tot_INTERACT Cortisolt Order
36	Proc_Asst Age Tot_INTERACT Testosteronet Order
37	Proc_Asst Age Wins Cortisolt Order
38	Proc_Asst Age Wins Testosteronet Order
39	Proc_Asst Weight2 Tot_INTERACT Cortisolt Order
40	Proc_Asst Weight2 Tot_INTERACT Testosteronet Order
41	Proc_Asst Weight2 Wins Cortisolt Order
42	Proc_Asst Weight2 Wins Testosteronet Order
43	Proc_Asst Wins Cortisolt Order
44	Proc_Asst Wins Testosteronet Order
45	Proc_Asst Wins Weight2 Testosteronet Order
46	Age
47	Aget
48	Weight2t
49	Dominance
50	Proc_NAsst Age Cortisolt Testosteronet
51	Proc_NAsst Age Number_attacked
52	Proc_NAsst Age Number_attacked Cortisolt
53	Proc_NAsst Age Number_attacked Testosteronet

54	Proc_NAsst Age Tot_INTERACT
55	Proc_NAsst Age Tot_INTERACT Cortisolt
56	Proc_NAsst Age Tot_INTERACT Testosteronet
57	Proc_NAsst Age Wins
58	Proc_NAsst Age Wins Cortisolt
59	Proc_NAsst Age Wins Testosteronet
60	Proc_NAsst Losses Weight2
61	Proc_NAsst Weight2 Wins
62	Proc_NAsst Weight2 Wins Cortisolt
63	Proc_NAsst Weight2 Losses
64	Proc_NAsst Weight2 Tot_INTERACT
65	Proc_NAsst Weight2 Tot_INTERACT Cortisolt
66	Proc_NAsst Weight2 Tot_INTERACT Testosteronet
67	Proc_NAsst Weight2 Wins
68	Proc_NAsst Weight2 Wins Cortisolt
69	Proc_NAsst Weight2 Wins Testosteronet
70	Proc_NAsst Wins
71	Proc_NAsst Wins Cortisolt
72	Proc_NAsst Wins Testosteronet
73	Proc_NAsst Wins Weight2
74	Proc_NAsst Wins Weight2 Cortisolt
75	Proc_NAsst Wins Weight2 Testosteronet
76	Proc_NAsst Age Cortisolt Testosteronet Order
77	Proc_NAsst Age Cortisolt*Testosteronet Order
78	Proc_NAsst Age Number_attacked Cortisolt Order
79	Proc_NAsst Age Number_attacked Testosteronet Order
80	Proc_NAsst Age Tot_INTERACT Cortisolt Order
81	Proc_NAsst Age Tot_INTERACT Testosteronet Order
82	Proc_NAsst Age Wins Cortisolt Order
83	Proc_NAsst Age Wins Testosteronet Order
84	Proc_NAsst Weight2 Tot_INTERACT Cortisolt Order
85	Proc_NAsst Weight2 Tot_INTERACT Testosteronet Order
86	Proc_NAsst Weight2 Wins Cortisolt Order
87	Proc_NAsst Weight2 Wins Testosteronet Order
88	Proc_NAsst Wins Cortisolt Order
89	Proc_NAsst Wins Testosteronet Order
90	Proc_NAsst Wins Weight2 Testosteronet Order
91	Null
